# Elevated levels of whole blood nickel in a group of Sri Lankan women with endometriosis: a case control study

**DOI:** 10.1186/1756-0500-6-13

**Published:** 2013-01-14

**Authors:** Nalinda Silva, Hemantha Senanayake, Vajira Waduge

**Affiliations:** 1 Lecturer in Physiology, Faculty of Medical Sciences University of Sri Jayewardenepura, Nugegoda, Sri Lanka; 2 Professor in Obstetrics and Gynecology, Department of Obstetrics and Gynecology, Faculty of Medicine, University of Colombo, Colombo, Sri Lanka; 3 Head of Life Sciences Division, Atomic Energy Authority, Colombo, Sri Lanka

**Keywords:** Endometriosis, Metalloestrogens, Nickel

## Abstract

**Background:**

Endometriosis is characterized by the persistence of endometrial tissue in ectopic sites outside the uterine cavity. Presence of nickel, cadmium and lead in ectopic endometrial tissue has been reported previously. While any association between blood levels of nickel and endometriosis is yet to be described in literature, conflicting reports are available with regards to cadmium and lead levels in blood and urine.

**Findings:**

In fifty patients with endometriosis and fifty age-matched controls confirmed by laparoscopy or laparotomy, whole blood samples were collected and digested using supra pure 65% HNO_3_. Whole blood levels of nickel and lead were measured using Total Reflection X-ray Fluorescence (TXRF) while cadmium levels were evaluated using graphite furnace atomic absorption spectroscopy (GFASS). Women with endometriosis had significantly higher (*P*=0^.^016) geometric mean (95% CI) whole blood nickel levels [2.6(1.9-3.3) μg/L] as compared to women without endometriosis [0.8 (0.7-0.9) μg/L]. Whole blood levels of cadmium and lead were similar between the two groups.

**Conclusions:**

Although women with endometriosis in this study population had higher levels of nickel in whole blood compared to controls, whether nickel could be considered as an aetiological factor in endometriosis remains inconclusive in view of the smaller sample that was evaluated.

## Findings

### Background

Endometriosis is an estrogen dependent chronic inflammatory disease characterized by the survival of endometrial tissue outside the uterine cavity [1]. Endometriosis which affects an estimated 6-10% of women of the reproductive age group, is a considerable burden to affected women, their families and healthcare systems [2].

Among the etiological factors that promote the survival of ectopic tissue, estrogenic compounds that promote protracted continuation these tissues are gaining prominence supported by persuasive evidence from *in vitro*, *in vivo* and human studies [3].

Compelling molecular [4], *in vitro*[5] and *in vivo*[6] evidence for the ability of metal ions to activate the estrogen receptor [7] has defined a group of xenoestrogens termed metalloestrogens [8]. While evidence for the purported estrogenicity of cadmium are in plenty, estrogenic properties of other metals such as nickel have also been demonstrated by some research groups [9].

*In vivo* experiments by Krugner-Higby et al. [10] have demonstrated that the occurrence of endometriosis was higher in six female rhesus monkeys exposed to a daily intake of 5 to 10 mg/Kg of lead as compared to a control group (n= 4) given lead-free water over a period of 19 years, but the difference was not statistically significant, probably due to the small sample size [10].

We have previously demonstrated the presence of cadmium, lead and nickel in ectopic endometrial tissue in a group of Sri Lankan women [11]. Heavy metals enter the human body either by ingestion or inhalation and are subsequently transferred to the vascular compartment (blood) [12]. Metals derived from the circulation get deposited in the tissues by various mechanisms [13]. We hypothesized the hematogenous route (blood) as a possible source of metals in the ectopic endometrial tissue.

The present study was conducted with the objective of exploring further the association between endometriosis and whole blood levels of cadmium, lead and nickel in the same group of women with endometriosis who were previously studied. We compared the whole blood levels of the cadmium, lead and nickel in women having endometriosis (in whom these metals were detected in ectopic endometrial tissue -cases) with the blood levels of same metals in women who had no evidence of endometriosis (controls).

## Methods

This case control study was conducted among women of the reproductive age group, at the Professorial Gynecology Unit of the National Hospital, Colombo, Sri Lanka. Patients awaiting elective laparotomy or laparoscopy for diagnostic and/or treatment purposes were included. Those who were diagnosed visually as having endometriosis subsequent to laparotomy or laparoscopy were selected as cases (n=50). Apart from the ectopic endometrial tissue sample that was collected as described in the previous study [11], simultaneous venous blood sample was collected from each case. Women matched for age in whom endometriosis had been excluded by laparoscopy or laparotomy were recruited as controls (n=50). Indications for laparoscopy or laparotomy in controls were subfertility, dysmenorrhea, chronic pelvic pain or detection of an ovarian mass in ultrasound scan.

A sample of venous blood was collected from all participants during intravenous cannulation at the time of induction of anesthesia into polypropelene tubes containing ethylene diamine tetraacetic acid (EDTA). Pre-operatively, informed written consent was obtained from all the participants. All the blood samples were stored in a −20°C freezer until analysis.

Whole blood samples were digested using a protocol described previously [14] with some modifications. Briefly, matched blood of cases and controls were allowed to reach the room temperature. Then whole blood samples were transferred to pre-treated, acid washed glass beakers. The wet weight of each sample was determined using a chemical balance. Each sample was prepared in duplicate. All the blood samples were digested using 65% supra pure Nitric acid (HNO_3_, Merck, USA) while maintaining a uniform temperature. The final solution was made in 2mL of 5% HNO_3_ that was prepared by diluting the 65% supra pure HNO_3_ with double distilled deionized water.

The total-reflection X-ray fluorescence (TXRF) machine available at the Atomic Energy Authority (AEA) of Sri Lanka was utilized to measure metals apart from cadmium. TXRF is a multi element analysis technique [15] capable of detecting an array of elements at detection levels of picograms per liter (pg/L) [15]. An internal standard, Gallium, allows quantification of metals using the Axil software. However, the TXRF that was used had an inherent weakness in measuring cadmium since it had a Molybdenum x-ray tube. Therefore, we used atomic absorption spectroscopy (model GBC 933AA) together with a graphite furnace (model GBC GF 3000) available at the Institute of Fundamental Studies (IFS), Kandy, Sri Lanka to estimate cadmium levels as described elsewhere[16]. Both AEA and IFS [16] are national level apex institutions in Sri Lanka that have international certifications for trace element analysis where regular quality assurance programs are conducted.

Quality control and validation were performed using reference material supplied by the International Atomic Energy Authority (IAEA-A-13), Seronorm™ trace elements in whole blood levels 1 (MR 4210) and National Institute of Standards & Technology (NIST) Gaithersburg, USA water sample with trace elements (SRM 1643e). For 96% of the determinations, repeatability error did not exceed 10%. The detection limits for nickel, lead and cadmium in whole blood were as follows 0.05, 1.0, 0.01 μg/L. The precision for nickel, lead and cadmium in the range of the samples analyzed in this study was + 2, 3 and 6%, respectively.

SPSS version 13 for Windows was used for statistical analysis. Log transformation of metal levels was done and means were compared using *t*-test.

Ethical clearance was obtained from the Ethical Review Committees of the Faculty of Medical Sciences, University of Sri Jayewardenepura and the National Hospital of Sri Lanka.

## Results and discussion

Mean (±SD) age in cases and controls were 33.0 (±5^.^4) and 32^.^7(±5^.^4) years respectively. Cases and controls were similar in body mass index while none of the women who participated in the study were current smokers. Other demographic, biological and dietary characteristics of this group of women with endometriosis have been described previously [17].

Cadmium, lead and nickel were detected in whole blood of all the participants. The whole blood nickel levels in cases were significantly higher compared to controls. Cases had lower whole blood cadmium levels and higher lead levels as compared to controls; however these differences were not statistically significant (Table 1).

**Table 1:** Levels of metalloestrogens in whole blood (μg/L) of cases and controls

**Metalloestrogen**	**Cases (n=50)**	**Controls (n=50)**	***P *value**†
Nickel	2.6 (1.9-3.3)	0.8 (0.7-0.9)	0.016
Lead	11.0 (8.6-13.3)	6.9 (5.7-8.0)	0.389
Cadmium	0.7 (0.7-0.9)	0.8 (0.6-1.0)	0.423

Data expressed as geometric mean (95% CI).

† *t*-test between blood levels of cases and controls.

Environmental pollution has been identified as a potential cause for the increase in the prevalence of endometriosis following industrialization [18]. Heavy metals are gaining prominence as pollutants that could affect human health profoundly, especially in the face of escalating levels of environmental contamination [19]. Women are susceptible to heavy metal toxicity due to differences in kinetics, mode of action, susceptibility and exposure [20,21].

The demonstration of higher levels of nickel in whole blood in women with endometriosis as compared to controls in our study is a novel finding. Hitherto, there is no published literature that has investigated the nexus between nickel and endometriosis. The whole blood nickel levels we are reporting are comparable to those reported from women in the general population in different countries [22,23]. Thus the levels we have detected are essentially non toxic levels although cases had higher nickel levels which may have contributed towards the presence of nickel in ectopic endometrial tissue.

Nickel similar to other heavy metals, primarily enters the human body either by ingestion or inhalation [24]. In addition, nickel is a common metal found in accessories frequently used by women [20] and its absorption via intact or damaged skin has been demonstrated [25]. Martin et al. [26] assessed the estrogenic potency of metals using the 50% effective concentration (EC_50_) of different metals as determined from dose–response curves performed in MCF-7 cells transiently transfected with the luciferase reporter construct. They found that nickel had a relative potency of 1.0 compared to estradiol [26] implying that nickel is a potent metalloestrogen.

Humans studies that attempted to elicit the association between cadmium and lead and endometriosis have yielded conflicting results. In a population based study in the United States, high blood concentrations of cadmium were associated with endometriosis [27]; while a study done in Belgium reported similar levels of cadmium and lead in blood in both cases and controls [28]. Urinary cadmium concentrations were similar in cases with endometriosis and controls [28,29]. The results of this study are in agreement with the previously published research apart from that of Jackson et al. In the study conducted by Jackson and co workers that probed the association between cadmium and endometriosis, a considerable number of women with endometriosis were smokers [27]. In contrast, in our cohort of women none were current smokers.

In conclusion in this group of women with endometriosis higher blood levels of nickel were observed. Whether nickel could be considered as an etiological factor in endometriosis remains inconclusive considering two factors, namely the small sample size evaluated in this study and the absence of a previous report of such a link. However based on the available scientific evidence for occupational and environmental exposure of women to metals including nickel, future research would prove to be invaluable in further exploring the association between nickel and endometriosis.

## Abbreviations

AEA: Atomic Energy Authority; EDTA: Ethylene diamine tetraacetic acid; IFS: Institute of Fundamental Studies; IAEA: International Atomic Energy Authority; NIST: National Institute of Standards & Technology; TXRF: Total-reflection X-ray fluorescence.

## Competing interests

Authors declare that they have no competing interests.

## Authors’ contributions

NS carried out the sample collection, analysis and drafted the manuscript. HS identified the study participants and carried out the surgical procedures. VW assisted in the TXRF analysis of the samples and interpreted the data. All authors read and approved the final manuscript.

## References

[B1] Donnez J Endometriosis: enigmatic in the pathogenesis and controversial in its therapy Fertil Steril 2012 98 3 509 510 10.1016/j.fertnstert.2012.07.1125 22938767

[B2] Markovic M Manderson L Warren N Endurance and contest: women's narratives of endometriosis Health (London) 2008 12 349 367 10.1177/1363459308090053 18579632

[B3] Garai J Molnar V Varga T Koppan M Torok A Bodis J Endometriosis: harmful survival of an ectopic tissue Front Biosci 2006 11 595 619 10.2741/1821 16146755

[B4] Stoica A Katzenellenbogen BS Martin MB Activation of estrogen receptor-alpha by the heavy metal cadmium Mol Endocrinol 2000 14 4 545 553 10.1210/me.14.4.545 10770491

[B5] Garcia-Morales P Aceda M Kenney N Kims N Salomon DS Gottardisn MM Solomonn HB Shollern PF Jordan C Martin MB Effect of cadmium on estrogen receptor levels and estrogen-induced responses in human breast cancer cells J Biol Chem 1994 269 24 16896 16901 8207012

[B6] Johnson MD Kenny N Stoica A Hilakivi-Clarke L Singh B Chepko G Clarke R Sholler PF Lirio AA Foss C Cadmium mimics the in vivo effects of estrogen in the uterus and mammary glands Nat Med 2003 9 8 1081 1084 10.1038/nm902 12858169

[B7] Safe S Cadmium disguise dupes the estrogen receptor Nat Med 2003 9 8 1000 1001 10.1038/nm0803-1000 12894163

[B8] Darbre PD Metalloestrogens: an emerging class of inorganic xenoestrogens with potential to add to the oestrogenic burden of the human breast J Appl Toxicol 2006 26 191 197 10.1002/jat.1135 16489580

[B9] Auino NB Sevigny MB Sabangan J Louie MC The role of cadmium and nickel in estrogen receptor signaling and breast cancer:metalloestrogens or not? J Environ Sci Health C Environ Carcinog Ecotoxicol Rev 2012 30 3 189 224 22970719 10.1080/10590501.2012.705159 PMC3476837

[B10] Krugner-Higby L Rosenstein A Handschke L Luck M Laughlin NK Mahvi D Gendron A Inguinal hernias, endometriosis, and other adverse outcomes in rhesus monkeys following lead exposure Neurotoxicol Teratol 2003 25 561 570 10.1016/S0892-0362(03)00076-X 12972069

[B11] Silva N Senanayake H Peiris-John R Wickremasinghe R Sathiakumar N Waduge V Presence of metalloestrogens in ectopic endometrial tissue J Pharmaceut Biomedsci 2012 24 24 1 5

[B12] Schweinberg F Von-Karsa L Heavy metal concentration in humans Comp Biochem Physiol 1990 95c 2 117 123 10.1016/0742-8413(90)90092-n 1977547

[B13] Bridges CC Zalups RK Molecular and ionic mimicry and the transport of toxic metals Toxicol Appl Pharmacol 2005 204 274 308 10.1016/j.taap.2004.09.007 15845419 PMC2409291

[B14] Nasiadek M Krawczyk T Sapotal A Tissue levels of cadmium and trace elements in patients with myoma and uterine cancer Hum Exp Toxicol 2005 24 623 630 10.1191/0960327105ht575oa 16408615

[B15] Wobrauschek P Total reflection x-ray fluorescence analysis—a review X Ray Spectrom 2007 36 289 300 10.1002/xrs.985

[B16] Bandara JMRS Senevirathna DMAN Dasanayake DMRSB Herath V Bandara JMRP Abeysekara T Rajapaksha KH Chronic renal failure among farm families in cascade irrigation systems in Sri Lanka associated with elevated dietary cadmium levels in rice and freshwater fish (Tilapia) Environ Geochem Health 2008 30 465 478 10.1007/s10653-007-9129-6 18200439

[B17] Silva N Senanayake H Peiris-John R Wickremasinghe R Demographic, biological and dietary characteristics associated with endometriosis in a group of Sri Lankan women Sri Lanka J Obstet Gynecol 2011 33 91 97

[B18] Heilier JF Donnez J Nackersd F Rousseaud R Verougstraetea V Rosenkranza K Donnezc O Grandjeanc F Lisona D Tongletd R Environmental and host-associated risk factors in endometriosis and deep endometriotic nodules: a matched case–control study Environ Res 2007 103 121 129 10.1016/j.envres.2006.04.004 16781705

[B19] World Health Organization Cadmium Environmental Health Criteria. Volume 134 1992 Geneva: IPCS -International Program on Chemical Safety

[B20] Butter ME Are women more vulnerable to environmental pollution? J Hum Ecol 2006 20 3 221 226

[B21] Vahter M A·kessona A Lide’n C Ceccatellia S Berglunda M Gender differences in the disposition and toxicity of metals Environ Res 2007 104 85 95 10.1016/j.envres.2006.08.003 16996054

[B22] Ikeda M Ohashi F Fukui Y Sakuragi S Moriguchi J Cadmium, chromium, lead, manganese and nickel concentrations in blood of women in non-polluted areas in Japan, as determined by inductively coupled plasma-sector field-mass spectrometry Int Arch Occup Environ Health 2011 84 2 139 150 10.1007/s00420-010-0542-2 20521063

[B23] Nunes JA Batistaa BL Rodriguesa JL Caldasb NM Netob JAG Barbosa F Simple method based on ICP-MS for estimation of background levels of arsenic, cadmium, copper, manganese, nickel, lead, and selenium in blood of the Brazilian population J Toxicol Environ Health A 2010 73 878 887 10.1080/15287391003744807 20563921

[B24] Agency for Toxic Substances and Disease Registry (ATSDR) Toxicological profile for Nickel 2005 39689221

[B25] Filon FL D’Agostin F Crosera M Adami G Bovenzi M Maina G In vitro absorption of metal powders through intact and damaged human skin Toxicol Vitr 2009 23 574 579 10.1016/j.tiv.2009.01.015 19490843

[B26] Martin MB Reiter R Pham T Avellet YR Camara J Lahm M Pentecost E Prathap K Gilmore BA Diverkar S Estrogen-like activity of metals in Mcf-7 breast cancer cells Endocrinology 2003 144 6 2425 2436 10.1210/en.2002-221054 12746304

[B27] Jackson LW Zullo MD Goldberg JM The association between heavy metals, endometriosis and uterine myomas among premenopausal women: National Health and Nutrition Examination Survey 1999–2002 Hum Reprod 2008 23 3 679 687 10.1093/humrep/dem394 18192673

[B28] Heilier JF Donnez J Verougstraete V Donnez O Grandjean F Haufroid V Nackers F Lison D Cadmium, lead and endometriosis Int Arch Occup Environ Health 2006 80 149 153 10.1007/s00420-006-0114-7 16688463

[B29] Itoh H Iwasaki M Nakajima Y Endo Y Hanaoka T Sasaki H Tanaka T Yang B Tsugane S A case–control study of the association between urinary cadmium concentration and endometriosis in infertile Japanese women Sci Total Environ 2008 402 171 175 10.1016/j.scitotenv.2008.05.006 18558423

